# Endovascular stentectomy using the snare over stent-retriever (SOS) technique: An experimental feasibility study

**DOI:** 10.1371/journal.pone.0178197

**Published:** 2017-05-25

**Authors:** Tareq Meyer, Omid Nikoubashman, Lisa Kabelitz, Marguerite Müller, Ahmed Othman, Saif Afat, Martin Kramer, Martin Wiesmann, Marc A. Brockmann, Carolin Brockmann

**Affiliations:** 1 Department of Diagnostic and Interventional Neuroradiology, University Hospital RWTH Aachen, Aachen, Germany; 2 Department of Neuroradiology, University Medical Centre of the Johannes Gutenberg University Mainz, Mainz, Germany; 3 Institute of Neuroscience and Medicine 4, Medical Imaging Physics, Forschungszentrum Jülich, Jülich, Germany; 4 Department of Veterinary Clinical Sciences, Small Animal Clinic, Justus-Liebig-University, Giessen, Germany; Universitatsklinikum Freiburg, GERMANY

## Abstract

Feasibility of endovascular stentectomy using a snare over stent-retriever (SOS) technique was evaluated in a silicon flow model and an in vivo swine model. In vitro, stentectomy of different intracranial stents using the SOS technique was feasible in 22 out of 24 (92%) retrieval maneuvers. In vivo, stentectomy was successful in 10 out of 10 procedures (100%). In one case self-limiting vasospasm was observed angiographically as a technique related complication in the animal model. Endovascular stentectomy using the SOS technique is feasible in an experimental setting and may be transferred to a clinical scenario.

## Introduction

Loss [1], dislocation [2], twisting, incomplete unfolding [3], as well as misplacement [4] of stents are complications encountered in interventional neuroradiology. Options to deal with these problems are limited and include surgical removal (either challenging in the neurovasculature or even impossible depending on the location) [2] [3], or placement of a second stent to fix the problem [4]. In many cases, however, the stent is left as it is and the patient is being continuosly treated with anti-platelet medication [5] carrying the risk of vessel stenosis, occlusion, and cerebral infarctions [6, 7].

In certain cases, stentectomy is therefore desirable. Devices like the Alligator retrieval device [5], the Merci retrieval system [8] or endovascular snares [5] have been used for stentectomy. Despite the tempting idea that forceps or snares are ideal for salvage, these tools were developed for peripheral interventions. Intracranial vessels differ from other vessels, as they have a smaller diameter, and lack a solid adherence to surrounding tissues and a thick tunica adventitia [9]. Hence, devices exerting greater forces to the vessel-wall may lead to periprocedural complications (e.g. perforation or dissection). Few case reports describe endovascular stentecetomy using snare retrieval alone [6, 10–12], pull-back maneuvers via balloon [1] or stent-based techniques [10, 11]. Recently, first abstracts also described the combination of a stent-retriever and a lasso for stentectomy [11].

Purpose of our experiments was to investigate the feasibility of using a combination of stent-retriever and lasso for stentectomy in an experimental setting.

## Materials and methods

### Study design

**Fig 1:**
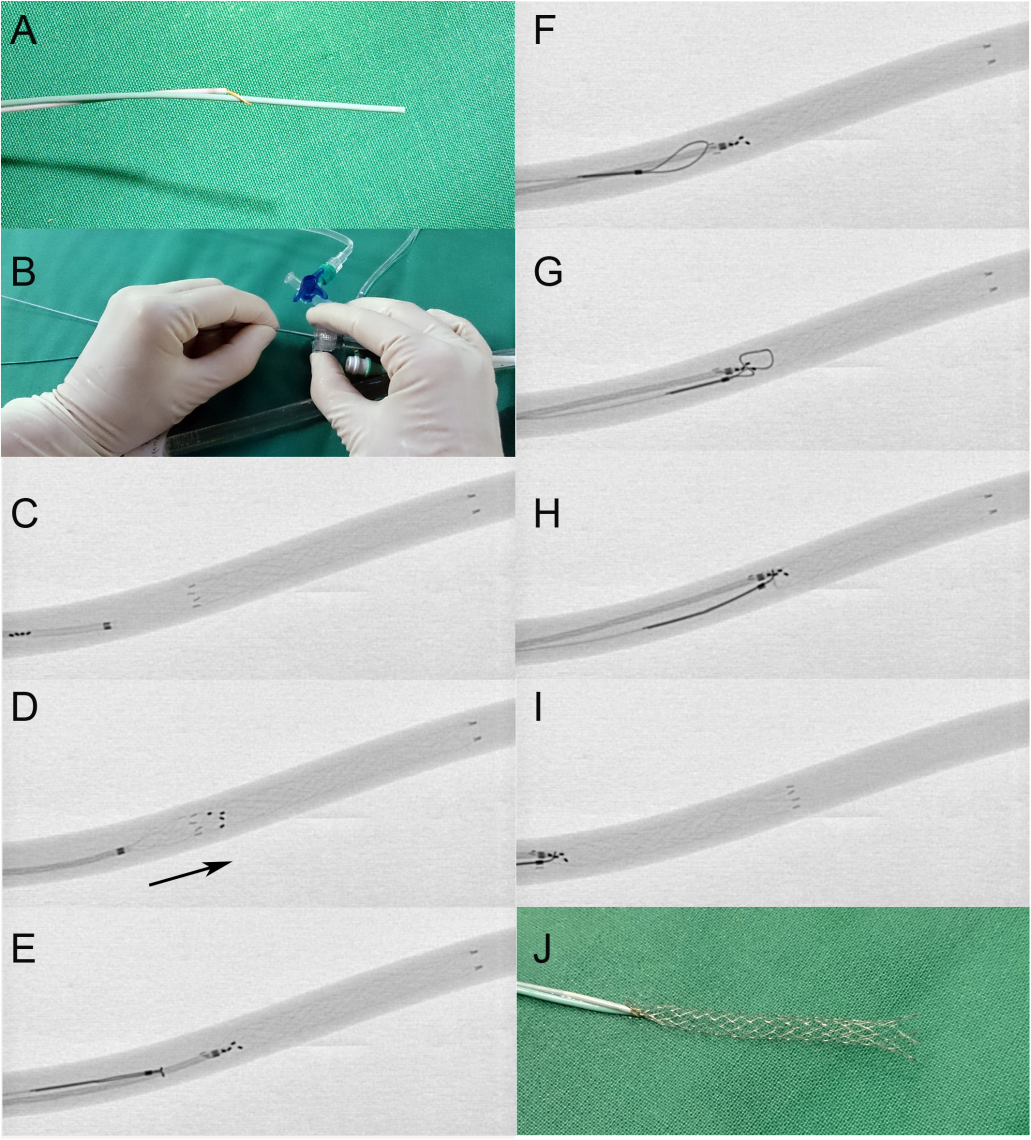
Stentectomy technique. 1.) The loop of a microsnare was slipped over the tip of a microcatheter, slightly tightened, (1A) and the microcatheter was inserted into a long sheath or guide catheter (1A) 2.) The microcatheter together with snare was positioned a few milimeters proximal of the mal-deployed stent (1C) 3.) A stent-retriever was advanced through the microcatheter and partially unfolded, so that the distal part of the stent-retriever attached to the vessel wall. 4.) The partially unfolded stent-retriever was pushed forward carefully, until the distal markers of the stent-retriever overlapped with the proximal markers of the mal-deployed stent (1D). Using a stent-retriever with a comparable or larger diameter than the target stent seems to be helpful. 5.) The stent-retriever is being resheathed while applying slight pressure, hereby „grabbing”the proximal end of the mal-deployed stent (1E). Doing so, some or all of the proximal markers of the maldeployed stent move towards the tip of the microcatheter (1E). 6.) The microsnare is being opened (1F), pushed forward over the microcatheter and over the proximal end of the maldeployed stent (1G). 7.) Finally, the microsnare is pulled close again (1H). 8.) The stent-retriever and the snare are „locked”in their position by tightening a torque on the wire of both devices directly adjacent to the hemostatic valve (not shown). 9.) To extract the maldeployed stent, the snare and the stent-retriever are being pulled back slowly into the sheath or guide catheter (1I) (aspirate to prevent embolism). 10.) If it should be impossible to pull a stent into the sheath or guide catheter, one should consider to “move” the stent into a less relevant vessel, like the external carotid artery or in a brachial or femoral vessel that can be reached more easily by a vascular surgeon.

Two experimental models were applied: 1. an in vitro silicon flow model and 2. an in vivo swine model. Stentectomy was tested 24 times in vitro and ten times in vivo [13]. Two different sizes of microsnare systems (4 and 7mm loop diameter) and two different sizes of stent-retrievers (4 x 20 mm and 6 x 30 mm) were applied to capture four different intracranial stents in each model. The procedure was carried out by three different operators: 1. ten years of experience (n = 15 runs in vitro, n = 8 runs in vivo), 2. one year of experience (n = 4 runs in vitro, n = 0 runs in vivo) and 3. no interventional experience (n = 5 runs in vitro, n = 2 runs in vivo). All operators were unexperienced regarding stentectomy, but trained the setup outside the flow model. In-vivo, the technique was likewise tested and angiographically controlled for possible complications. Descriptive statistics were applied. Stentectomy was defined as successful if the stent could be extracted into the guide catheter at first attempt. Loss of a stent during extraction or inability to “grab” the stent for extraction were defined as unsuccessful attempts. The technique used for stentectomy is described and visualized in detail in Fig 1 and additionally in two supplementary movie files (S1 and S2 Movies) available online.

### In vitro flow model

A circulatory silicon flow model was used for in-vitro experiments [14]. A programmable roller pump (MCP V5.12; ISMATEC, Glattbrugg, Switzerland) was set to imitate a physiologic blood flow (pulse frequency 86 bpm; flow volume 286 mL/min). As human blood substitute a translucent non-Newtonian aqueous glycerol solution was used (40% glycerin (Glycerin 98%; Carl Roth GmbH, Karlsruhe, Germany) and destilled water) with a bloodlike viscosity of 3.75 mPA/s at room temperature. Different tube-diameters from which the stents were extracted were used simulating different vessel sizes (4.5 mm, n = 4; 2.5 mm, n = 10; 4.3 mm, n = 10). The following three stent-retrievers (frequently used in clinical settings and arbitrarily available for experimental purpose in our department) were used for stentectomy: 3 x 20 mm Trevo XP ProVue (Stryker; Kalamazoo, MI, USA), 4 mm x 20 mm and 6 mm x 30 mm Solitaire FR (Covidien/Ev3; Irvine, CA, USA). The following four stents (likewise commonly used in clinical routine and available for experimental purpose in our department) were implanted and extracted: 1. Enterprise stent (Cordis Neurovascular; Miami, FL, USA), 2. Pipeline Embolization Device (PED) (Covidien/Ev3; Irvine, CA, USA), 3. Flow Re-Direction Endoluminal Device flow diverter (FRED) (MicroVention; Tustin, CA, USA) and 4. Acclino flex (ACANDIS GmbH & Co. KG, Pforzheim, Germany). Two microsnare systems (4 mm and 7 mm; Amplatz GooseNeck Microsnare (Covidien/EV3; Irvine, CA, USA)) were used for fixation (Table 1). The stent-retrievers were advanced through a Trevo 18 MC microcatheter (Stryker) or a Rebar 18 microcatheter (Covidien/Ev3). Stents were extracted into an 8F Guider Softip catheter (Boston Scientific; Marlborough, MA, USA) (S1 and S2 Movies).

**Table 1:** In vitro procedures.

*n*	*Device used for stent extraction*	*Salvaged stent*	*Vessel diameter*	*Experience interventionalist*	*Procedure comments*	*Success*
***1***	Solitaire FR 6 x 30 mm	Enterprise 4.5 x 22 mm	4.5 mm	++	—	✓
	Amplatz GooseNeck Microsnare 7 mm					
***2***	Solitaire FR 6 x 30 mm	Enterprise 4.5 x 22 mm	4.5 mm	+++	Stent-retriever imposed on Enterprise after 2nd try	✓
	Amplatz GooseNeck Microsnare 7 mm					
***3***	Solitaire FR 6 x 30 mm	Enterprise 4.5 x 22 mm	4.5 mm	+	—	✓
	Amplatz GooseNeck Microsnare 7 mm					
***4***	Solitaire FR 6 x 30 mm	Enterprise 4.5 x 22 mm	4.5 mm	++	Stent-retriever imposed on Enterprise after 2nd try	✓
	Amplatz GooseNeck Microsnare 4 mm					
***5***	Solitaire FR 6 x 30 mm	Enterprise 4.5 x 22 mm	2.5 mm	++	—	✓
	Amplatz GooseNeck Microsnare 4 mm					
***6***	Solitaire FR 6 x 30 mm	Enterprise 4.5 x 22 mm	2.5 mm	+++	—	✓
	Amplatz GooseNeck Microsnare 4 mm					
***7***	Solitaire FR 6 x 30 mm	Enterprise 4.5 x 22 mm	2.5 mm	+++	—	✓
	Amplatz GooseNeck Microsnare 4 mm					
***8***	Solitaire FR 6 x 30 mm	Enterprise 4.5 x 22 mm	2.5 mm	+	—	✓
	Amplatz GooseNeck Microsnare 4 mm					
***9***	Solitaire FR 6 x 30 mm	PED 4.5 x 25 mm	2.5 mm	+++	PED lost during extraction	×
	Amplatz GooseNeck Microsnare 4 mm					
***10***	Trevo XP ProVue 3 x 20 mm	PED 4.5 x 25 mm	2.5 mm	+++	Stent-retriever did not interlock with PED	×
	Amplatz GooseNeck Microsnare 4 mm					
***11***	Solitaire FR 4 x 20 mm	PED 4.5 x 25 mm	2.5 mm	+++	—	✓
	Amplatz GooseNeck Microsnare 4 mm					
***12***	Solitaire FR 4 x 20 mm	PED 4.5 x 25 mm	2.5 mm	+++	—	✓
	Amplatz GooseNeck Microsnare 4 mm					
***13***	Solitaire FR 4 x 20 mm	FRED 5.5 x 25 mm	2.5 mm	+++	—	✓
	Amplatz GooseNeck Microsnare 4 mm					
***14***	Solitaire FR 4 x 20 mm	FRED 5.5 x 25 mm	2.5 mm	+	—	✓
	Amplatz GooseNeck Microsnare 4 mm					
***15***	Solitaire FR 6 x 30 mm	Acclino 4.5 x 22 mm	4.3 mm	+++	—	✓
	Amplatz GooseNeck Microsnare 4 mm					
***16***	Solitaire FR 6 x 30 mm	Acclino 4.5 x 22 mm	4.3 mm	++	—	✓
	Amplatz GooseNeck Microsnare 4 mm					
***17***	Solitaire FR 6 x 30 mm	Acclino 4.5 x 22 mm	4.3 mm	++	—	✓
	Amplatz GooseNeck Microsnare 4 mm					
***18***	Solitaire FR 6 x 30 mm	Acclino 4.5 x 22 mm	4.3 mm	+++	—	✓
	Amplatz GooseNeck Microsnare 4 mm					
***19***	Solitaire FR 6 x 30 mm	Acclino 4.5 x 22 mm	4.3 mm	+++	—	✓
	Amplatz GooseNeck Microsnare 4 mm					
***20***	Solitaire FR 6 x 30 mm	Acclino 4.5 x 22 mm	4.3 mm	+	—	✓
	Amplatz GooseNeck Microsnare 4 mm					
***21***	Solitaire FR 6 x 30 mm	Acclino 4.5 x 22 mm	4.3 mm	+++	—	✓
	Amplatz GooseNeck Microsnare 4 mm					
***22***	Solitaire FR 6 x 30 mm	Acclino 4.5 x 22 mm	4.3 mm	+++	—	✓
	Amplatz GooseNeck Microsnare 4 mm					
***23***	Solitaire FR 6 x 30 mm	Acclino 4.5 x 22 mm	4.3 mm	+++	—	✓
	Amplatz GooseNeck Microsnare 4 mm					
***24***	Solitaire FR 6 x 30 mm	Acclino 4.5 x 22 mm	4.3 mm	+++	—	✓
	Amplatz GooseNeck Microsnare 4 mm					

### In vivo swine model

The animal trial using female German Landrace swines (weight approximately 70 kg ± 5 kg) was approved by the institutional animal care board and the responsible State Agency (LANUV–Landesamt für Natur, Umwelt und Verbraucherschutz Nordrhein-Westfalen). The animals were raised and housed by an extern breeder and were transported to the clinic shortly before the experiments. Atropine (1.5 ml, 1%; Atropin), azaperone (0.1 ml / kg; Stresnil) and ketamine (0.1 ml / kg, 10%; Ketamin) were used for premedication, followed by intubation and mechanical ventilation with an oxygen-air mixture. Propofol (2%, 8–12 mg / kg / hour), fentanyl (45–90 μg / kg / hour) and phenobarbital (160 mg / ml; Narcoren) were used for maintaining the anesthesia. All animals received heparin (5000 IU) and acetylsalicylic acid (500 mg; Aspirin) intravenously after arterial puncture. To prevent dehydration, constant saline infusion was performed. The stents were employed in healthy forelimb-arteries. Intracranial experiments were not possible as swine have a rete mirabile preventing access to the cerebral circulation. Two different stent-retrievers (4 x 20 mm Trevo XP ProVue (Stryker), 4.0 x 20 mm Solitaire FR (Covidien/Ev3) and two microsnare systems (4.0 mm and 7.0 mm Amplatz GooseNeck Microsnare (Covidien/EV3)) were used for stentectomy of four different types of stent: 1.) 4.5 mm x 28 mm Enterprise (Cordis Neurovascular), 2.) Neuroform Atlas 4.0 x 24 mm (Stryker), 3. + 4.) 2.5 x 20 and 2.75 x 20 Pharos.

Vitesse (Codman Neuro; Raynham, MA, USA) (Table 2). To simulate the loss of incompletely opened balloon-mounted stents, the balloon of the Pharos Vitesse stent was minimally inflated, then deflated, and finally the stent was stripped off at the tip of the guide catheter. The stent-retrievers were advanced through a Trevo 18 MC micro-catheter (Stryker) or a Rebar 18 microcatheter (Covidien/Ev3). The stents were extracted into an 8F Guider Softip guiding catheter (Boston Scientific; Marlborough, MA, USA) or into a 9F Merci Balloon.

**Table 2:** In vivo procedures.

*n*	*Device used for stent extraction*	*Salvaged stent*	*Vessel diameter*	*Experience interventionalist*	*Procedure comments*	*Success*
***1***	Trevo XP ProVue 4 x 20 mm	Enterprise 4.5 x 28 mm	2.7 mm	+	—	✓
	Amplatz GooseNeck Microsnare 7 mm					
***2***	Trevo XP ProVue 4 x 20 mm	Enterprise 4.5 x 28 mm	3.3 mm	+++	—	✓
	Amplatz GooseNeck Microsnare 7 mm					
***3***	Trevo XP ProVue 4 x 20 mm	Enterprise 4.5 x 28 mm	2.6 mm	+	—	✓
	Amplatz GooseNeck Microsnare 7 mm					
***4***	Trevo XP ProVue 4 x 20 mm	Enterprise 4.5 x 28 mm	2.3 mm	+++	self limiting vasospasm	✓
	Amplatz GooseNeck Microsnare 4 mm					
***5***	Solitaire FR 4 x 20 mm	Pharos Vitesse incompletely inflated	2.2 mm	+++	—	✓
	Amplatz GooseNeck Microsnare 4 mm					
***6***	Solitaire FR 4 x 20 mm	Pharos Vitesse incompletely inflated	2.1 mm	+++	—	✓
	Amplatz GooseNeck Microsnare 4 mm					
***7***	Solitaire FR 4 x 20 mm	Pharos Vitesse incompletely inflated	2.4 mm	+++	—	✓
	Amplatz GooseNeck Microsnare 4 mm					
***8***	Solitaire FR 4 x 20 mm	Pharos Vitesse incompletely inflated	2.9 mm	+++	—	✓
	Amplatz GooseNeck Microsnare 4 mm					
***9***	Solitaire FR 4 x 20 mm	Pharos Vitesse incompletely inflated	2.8 mm	+++	—	✓
	Amplatz GooseNeck Microsnare 4 mm					
***10***	Trevo XP ProVue 4 x 20 mm	Neuroform Atlas 4.0 x 24 mm	3.2 mm	+++	—	✓
	Amplatz GooseNeck Microsnare 4 mm					

Guide Catheter (Stryker). After carrying out all trials, the animals were euthanized by a veterinarian.

## Results

### In vitro experiments

The overall success rate was 22 of 24 cases (92%) (Table 1). Two failures were encountered by the experienced operator trying to retrieve the PED from the in vitro model. Once, the stent was lost during retraction into the sheath (probably due to insufficient fixation by stent and microsnare). In the other case “grabbing” the proximal end of the PED failed. In two further attempts PED-extraction was successful, although again “grabbing” was difficult (S1 movie).

### In vivo experiments

Stentectomy of self-expanding stents (Enterprise; Cordis) was feasible in five out of five cases (Table 2). In one case a self-limiting vasospasm was observed. Stentectomy of an open-cell design stent (Neuroform Atlas; Stryker) was successful (n = 1) without complication despite its design. Incompletely inflated Pharos Vitesse stents (n = 5) likewise were salvaged without any complications. Success of the SOS-technique was independend of the operators experience.

## Discussion

Stentectomy has been described in the literature by several authors. Miley et al. used a pull-back technique with a balloon in one patient [1]. Castano et al. were successful in 3 out of 6 cases by pure snare retrieval as long as the proximal markers did not strike the artery wall in an unfavourable angle [12] and if the markers were visualized well. Parthasarathy et al. describe the succesful use of a stent-retriever-based technique (deploy-and-engage technique) in one patient and a loop-and-snare technique in another patient [10]. The authors report that pure stent-based retrieval may be limited by a weak hold onto the displaced or detached stent during retrieval [10]. The only large clinical series of stentectomy has been reported by Chapot et al. (unpublished results / oral presentation at the ALICE 2015). In their series stentectomy was successful in 26 out of 28 patients using different techniques. They reported the technique (as described in the underlying manuscript) to be successful in 9 out of 10 patients. Thus, our experimental results match well with the clinical experience of Chapot et al. and show that the SOS technique is an efficient method for stentectomy.

The SOS technique was developed for situations where isolated devices did not enable to extract a self-expandable stent whereas the combination of these devices enabled to do so. We have been able to confirm these problems in our experiments: Using a stent-retriever alone without additional fixation (i.e. lasso) during retrieval we frequently lost stents (in our earlier experiments).

Another interesting technique is the “loop-and-snare technique“, [10] which likewise should provide sufficient stability during retrieval: Briefly, a microwire is being advanced through the mesh of a mal-deployed stent. The microwire is then being captured using a snare and the stent extracted. The technique was successfully used in one case by Parthasarathy et al.

The major limitation of our study is the experimental setup. Thus, the extreme tortuosity of cerebral vessels and the possibility of cerebral vessel injury, arterial occlusion, clot formation or plaque embolization were not evaluated in real patients. Therefore the study to some degree is lacking generalizibility of the experimental success. On the other hand, in pigs we did not observe relevant vessel injury although admitetly histologic correlation was not made. Furthermore, others report successful retrieval maneuvers from the cerebral circulation[10, 11]. Another limitation is the reduced number of operators. However, we also trained this technique in a workshop some months ago and found that almost all of the participants were able to successfully use the SOS technique.

Only a limited number of different devices were tested. The rationale for this is simply that most of these devices were available in our department. However, we suppose that most other stent-retrievers from other companies will generate comparable results (as long as they feature a similar design without a distal tip). The retrieved stents were available for experimental purpose in our department and we do not see any reasons why this rescue technique should not work for other similar stents. The number of iterations performed was arbitrary. Nevertheless, the findings indicate that the snare over stent-retriever (SOS) technique is feasible for endovascular salvage of intracranial stents.

## Supporting information

S1 Movie PED extraction. This film shows stentectomy of a Pipeline Embolization Device (PED). During the first attempts, it is difficult to envelop the PED with the distal markers of the stent-retriever. After successfully envelopping the PED, the snare is opened and set tight again over the overlapping section of both. Now, by pulling simultaneously on stent-retriever and snare, the PED elongates before following the traction of stent-retriever and snare towards the sheath.
(MOV)

S2 Movie FRED extraction. This film visualizes stentectomy of a Flow Redirection Endoluminal Device Stent System (FRED). The stent-retriever is able to envelop the FRED at first attempt. The stent-retriever is resheathed while applying slight pressure resulting in an interlock of stent-retriever and FRED. Afterwards, the snare is opened and pushed forward towards the overlapping section of both stents. It takes a few trials until the microsnare is positioned correctly. Now the microsnare is set tight and all components can be pulled towards the sheath.
(MOV)
